# Suggestions Relating to the Examination of the Teeth

**Published:** 1904-03

**Authors:** Charles G. Pike


### SUGGESTIONS RELATING TO THE EXAMINATION OF THE TEETH.[1]

BY CHARLES G. PIKE, D.M.D.

Can any one tell me how one may derive more benefit and pleasure than by gazing upon something that is of special interest to him. For instance, of course you all remember your first visit from home, how carefully and critically you watched everything, and on your return you spent much time in talking and thinking to yourself of the many beautiful and wonderful things which you had seen. How improving, how lasting were those first impressions! I am sure you will never forget them as long as you live.

Now, gentlemen, a number of months—yes, years—have passed since we first travelled or looked over this ground, and in the intervening time it has been our custom to travel, or, better, see it daily. Are we as critical and careful in our observations as we were on our first examination of the object, or whatever it might be? I am afraid we do not pay as marked attention as we should.

Now, do you suppose that there is any one present that has forgotten his first patient, the first examination that he was called upon to make? How carefully, observingly you went about it, not failing to ask many questions, many no doubt entirely uncalled

---

[1] Read before the Harvard Odontological Society, September 24, 1903.

for. In all probability you had taken at least a good half-hour, if not longer, in making your first examination.

How many of us now spend as much time? Would not our diagnosis of a case be more successful not only to ourselves, but to our patients, if before remarking about a filling or the treatment of a certain case we gave it a few moments careful thought? How quickly a patient will question us, and especially if we have made a statement that would have been much better unsaid. In order to make a proper examination, of course the teeth should be thoroughly cleansed, but this may not always be necessary.

A perfect examination, in my opinion, is not an easy thing to make. I do not mean that we are not all capable of doing it properly, but simply that we must give it as much care and attention as any filling that we put in. Time is a very important factor, and should always be considered. How often you will have patients say that they have not been satisfied with the operation or opinions of others, and ask at the same time a careful examination of their teeth.

In making an examination I have always made it a plan to first dry the teeth as much as possible with napkins and rolls and then proceed to examine, beginning at the superior right third molar, or as near it as is possible if such teeth are lacking, exploring with any suitable explorer every surface before passing to the next.

It is especially wise to explore every fissure and groove very carefully, never relying on the eyes for a decision, for often, on exploring in this manner, you will find spots which upon being broken down are found to be quite large and could have been detected in no other way.

After following these directions with each tooth of the superior arch, I then take silk and make as careful an examination as I can of the approximal surfaces. If there is the slightest doubt in my mind about the condition of any of these surfaces I suggest a wedge. I am a great believer in wedges, and know of nothing that can be used, if used with ordinary precautions, with less discomfort to the patient or better advantage to the operator. It seems to me to be the only correct way of examining doubtful approximal surfaces. If there is the least particle of doubt, a small wedge for a few hours will place you on a firm foundation, and that is what we must have in order to do satisfactory work.

Now, the question that some will ask is, Are the patients willing to allow this wedging? In some cases I will say that they will object at first, but after spending a few moments explaining the importance of it they will readily consent, and I am sure that after the first examination they will see for themselves the importance of it and you will never be criticised thereafter.

Oftentimes your patient will get so accustomed to rely upon the wedge for a diagnosis that before making their semiannual or annual visit to you, as the case may be, they will of their own accord insert wedging in doubtful places, so that if there is any trouble it can be detected at once.

In regard to a fee for this work, is there anything we do that requires any more attention on our part? If we take ten or twenty minutes for a small operation on a patient we expect a suitable fee, then why should not we expect the same from a patient after we have made an examination and pronounced the mouth in good condition, if such is the case. It is quite as much an operation as anything we do, and we should certainly receive proper remuneration for it.

Most any chart is suitable for an examination record, but a chart record should certainly be kept of each mouth for reference at any time. It is of special value to the operator in saving time, for by a hasty examination of it, just before the appointment, he can readily see what there is to be done and be ready to begin upon the arrival of the patient. Personally I use them to make notes on in regard to the patient or anything that is of special importance to me.

In closing, gentlemen, let me state that if there are any present who have at times allowed themselves to be lax in this apparently simple operation, I hope from now on they will try and make this one of their ideals and objects in their profession, for in order to attain the height for which we are all striving we must do our best in all things.

There occurred this short article in one of the recent publications of the *Dental Cosmos,* which, with your kind attention and permission, I will quote. Its subject is "Purpose in Life."

"Whoever desires to cultivate his firmness of purpose does well to begin with this leading one: He must have a definite aim, and keep it strictly in view; to it he must steadily direct his efforts and apply his powers. If he does this he will soon acquire a facility in its details which can come in no other way, and his interest in it will increase, for we always like to do that which we can do well. Of course, whether or not he will actually attain to that on which he sets his mind must depend upon the aim itself.

"He may aspire to be a great artist or statesman or lawyer or architect, and never be able to reach the goal, but his persistence will enable him to come as near the mark as his powers will allow, which is all that any man can do; whereas, with tenfold the ability, but with no steadfast purpose, he would have fallen far short of the place he now occupies.

"Success in its best sense is not always found in attaining that for which we plan; but rather in making the best that can be made of ourselves, doing as well and rising as high as our powers will allow. As the poet Young has well said,—

"'Thy purpose firm is equal to the deed.
Who does the best his circumstance allows,
Does well, acts nobly; angels could no more.'"

